# Suppressed humoral immunity is associated with dengue nonstructural protein NS1-elicited anti-death receptor antibody fractions in mice

**DOI:** 10.1038/s41598-020-62958-0

**Published:** 2020-04-14

**Authors:** Chung-Lin Tsai, Der-Shan Sun, Mei-Tzu Su, Te-Sheng Lien, Yen-Hsu Chen, Chun-Yu Lin, Chung-Hao Huang, Chwan-Chuen King, Chen-Ru Li, Tai-Hung Chen, Yu-Hsiang Chiu, Chun-Chi Lu, Hsin-Hou Chang

**Affiliations:** 1 Department of Molecular Biology and Human Genetics, Tzu-Chi University, Hualien, Taiwan; 2 Department of Internal Medicine, Kaohsiung Municipal Ta-Tung Hospital, Kaohsiung, Taiwan; 3 School of Medicine, Graduate Institute of Medicine, Sepsis Research Center, Center of Tropical Medicine and Infectious diseases, Kaohsiung Medical University, Kaohsiung, Taiwan; 4 Department of Biological Science and Technology, College of Biological Science and Technology, National Chiao Tung University, HsinChu, Taiwan; 5 Division of Infectious Diseases, Department of Internal Medicine, Kaohsiung Medical University Hospital, Kaohsiung Medical University, Kaohsiung, Taiwan; 6 Institute of Epidemiology and Preventive Medicine, National Taiwan University, Taipei, Taiwan; 7 Division of Rheumatology/Immunology and Allergy, Department of Internal Medicine, Tri-Service General Hospital, National Defense Medical Center, Taipei, Taiwan; 8 Present Address: Department of Experimental Immunology, Institute of Development, Aging and Cancer, Tohoku University, Sendai, Japan

**Keywords:** Viral infection, Antibodies

## Abstract

Dengue virus (DENV) infections may cause life-threatening dengue hemorrhagic fever (DHF). Suppressed protective immunity was shown in these patients. Although several hypotheses have been formulated, the mechanism of DENV-induced immunosuppression remains unclear. Previously, we found that cross-reactive antibodies against tumor necrosis factor-related apoptosis-inducing ligand (TRAIL) receptor 1 (death receptor 4 [DR4]) were elicited in DHF patients, and that anti-DR4 autoantibody fractions were elicited by nonstructural protein 1 (NS1) immunizations in experimental mice. In this study, we found that anti-DR4 antibodies could suppress B lymphocyte function *in vitro* and *in vivo*. Treatment with the anti-DR4 immunoglobulin (Ig) induced caspase-dependent cell death in immortalized B lymphocyte Raji cells *in vitro*. Anti-DR4 Igs elicited by NS1 and DR4 immunizations markedly suppressed mouse spleen transitional T2 B (IgM^+^IgD^+^), bone marrow pre-pro-B (B220^+^CD43^+^), pre-B (B220^+^CD43^−^), and mature B cell (B220^+^IgD^+^) subsets in mice. Furthermore, functional analysis revealed that the pre-elicitation of anti-NS1 and anti-DR4 Ig titers suppressed subsequently neutralizing antibody production by immunization with DENV envelop protein. Our data suggest that the elicitation of anti-DR4 titers through DENV NS1 immunization plays a suppressive role in humoral immunity in mice.

## Introduction

The dengue virus (DENV) is a mosquito-borne single positive-stranded RNA virus belonging to the *Flaviviridae* family (genus *Flavivirus*); the 4 major serotypes of DENV cause self-limiting dengue fever (DF) and life-threatening dengue hemorrhagic fever (DHF)^1^. It has been estimated that 50 million cases of DENV infections occur, and approximately 500,000 patients have been hospitalized with DHF, mainly in tropical and subtropical regions^2^. Evidence has suggested that because of the geographical extension of DENV infection and increases in the number of DENV cases and disease severity, DF and DHF have become major public health problems, with more than one-third of the global population residing in high-transmission-risk areas^3–5^. DHF is a complex disease, and its mechanism remains elusive. Currently, no specific treatment or effective vaccine is available for DHF^4^. Because prior DENV infection is a risk factor for developing DHF during secondary infections^5^, an abnormal immune response has been considered a vital component of the DHF pathophysiology.

Various studies have reported immunosuppression in patients with severe DENV infections^6^; while little is known about how the immune system is disrupted. Several hypotheses have been formulated to explain this phenomenon^6–8^ For example, according to the widely accepted hypothesis of antibody-dependent enhancement (ADE), in the initial (first) step, instead of providing protective immunity, preexisting nonneutralizing anti-DENV antibodies can form immune complexes with DENVs, substantially increasing the infectivity of DENVs targeting Fc receptor-expressing leukocytes^9–11^. It is suggested that DENV antibody-complexes boost virus production per infected cell by suppressing intracellular antiviral responses^6,7,12^. Accordingly, ADE of DENV secondarily leads to an increased overall virus replication in the leukocytes and a suppressed leukocyte-mediated immunity in the following (second and later) steps. Because the nonneutralizing anti-DENV antibodies are elicited before ADE, additional explanation regarding how DENV infections lead to immunosuppression in the first step that prior to ADE is required. One hypothesis, original antigenic sin, suggests that initial immunosuppression occurs before ADE^13,14^. However, the detailed mechanism that leads to the production of nonneutralizing antibodies and particularly the relevant B cell response remains elusive.

Clinical^15–17^, cellular, and animal studies^18–26^ have found that DENV infections and DENV nonstructural protein 1 (NS1) immunizations elicit autoantibodies against plasma, platelet, and endothelial antigens. Among these autoantigens, our previous report suggest that endothelial TNF-related apoptosis-inducing ligand (TRAIL) receptor 1 (death receptor 4 [DR4]; also known as TNFRSF10A) could be a potential autoantibody target leading to plasma leakage in severe DENV infections in our previous study^23^. DR4 is a member of the TNF receptor (TNFR) family and shows certain structural similarities with the TNFR family members of TACI, BCMA, and BAFFR. If DENV also elicits cross-reactive autoantibodies targeting to TACI, BCMA, and BAFFR, the lymphocyte regulation and particularly B cell function may therefore be impaired. However, this possibility is not yet verified. As a result, in this study, we hypothesize that in DENV infections, autoantibodies may be generated against 3 receptors of B-cell activating factor (BAFF; also known as BLyS and TNFSF13B), which include B-cell maturation antigen (BCMA; also known as TNFRSF17), transmembrane activator and CAML interactor (TACI; also known as TNFRSF13B) and BAFF receptor 3 (BAFFR; also known as TNFRSF13C). BAFF is a cytokine that belongs to the tumor necrosis factor (TNF) ligand family and is mainly expressed on B lymphocytes, and BAFF expression varies depending on B cell maturation. All 3 BAFF receptors, TACI, BCMA, and BAFFR, are expressed on B cells. TACI is found on a subset of T cells, and BCMA is found on plasma cells. These receptors play critical roles in regulating B lymphocyte proliferation, differentiation, maturation, and activation^27–29^. Collectively, the involvement of TACI, BCMA, and BAFFR in autoantibody mediated pathogenesis in dengue may be worth of further investigations.

Therefore, to extend our previous experiment, in this study, we investigated whether the serum samples of DHF patients and DENV NS1-immunized rabbits contain immunoglobulin (Ig) fractions against TACI, BCMA, and BAFFR. Moreover, we analyzed the association of the induction of anti-death receptor autoantibody fractions with the lymphocyte populations and neutralizing antibody production using a mouse model and discuss the potential immunosuppressive mechanism.

## Results

### Anti-TACI antibodies were detected in DHF patients and DENV NS1-immunized rabbits

**Figure 1:**
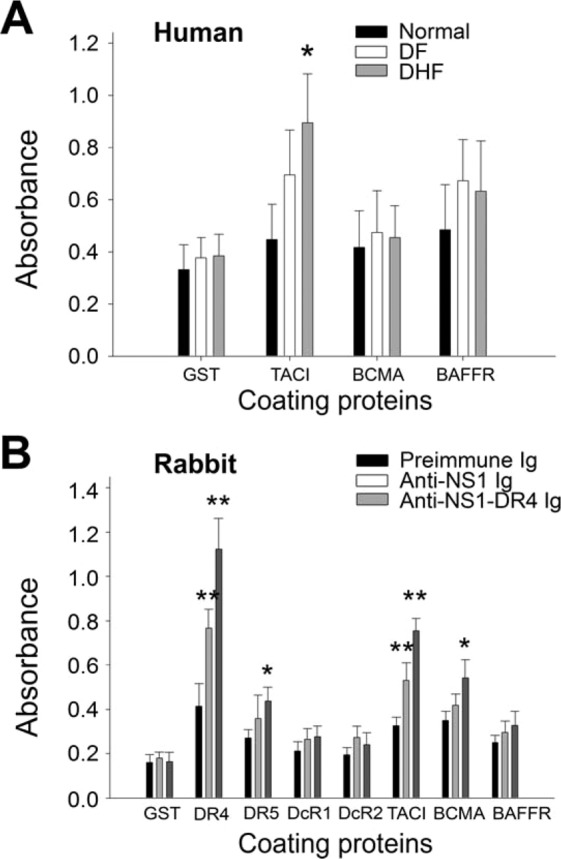
Detection of Ig titers against TACI, BCMA and BAFFR using ELISA. Normal donors- and patients with DF and DHF-derived IgG titers, which bound to microtiter plates that coated with GST and TNF-receptor family proteins, TACI, BCMA and BAFFR (**A)**. Cross reactivity of anti-NS1 Ig and anti-NS1-DR4 Ig toward TNF-receptor family proteins, are showed (**B**). Anti-NS1-DR4 Ig: DR4 affinity-column-purified Ig fraction from the anti-NS1 polyclonal Ig. *P < 0.05, **P < 0.01, comparisons to respective normal groups (**A**) (normal n = 12, DF n = 21, DHF n = 14), and respective preimmune Ig groups (**B**) (n = 6, 3 experiments with 2 replicates).

In our previous study, we found anti-TRAIL receptor (DR4; TNFRSF10A) antibody titers in the serum samples of DHF patients and DENV NS1-immunized rabbits^23^. Because death receptor families share structural similarity among members, in this study, we investigated whether antibody fractions against TACI, BCMA, and BAFFR are present in the serum samples of DHF patients and DENV NS1-immunized rabbits. We analyzed the anti-TACI, anti-BCMA, and anti-BAFFR Ig levels in patients infected with DENV using enzyme-linked immunosorbent assay (ELISA). The results revealed that patients with DHF tended to exhibit higher IgG titers against TACI compared with normal donors and patients with DF (Fig. 1A). Rabbit Igs were used to investigate whether DENV NS1 immunization elicits anti-TACI titers. Consistent with our previous study, ELISA data showed that anti-DR4 titers were elicited following immunization with recombinant NS1 (rNS1) (Fig. 1B). Notably, both the anti-NS1 Ig and a DR4 affinity-enriched fraction of the anti-NS1 Ig (anti-NS1-DR4 Ig) could still recognize TACI substrates (Fig. 1B, TACI group).

### Anti-TACI and anti-DR4 antibodies induced cell death in lymphoid cell lines

**Figure 2:**
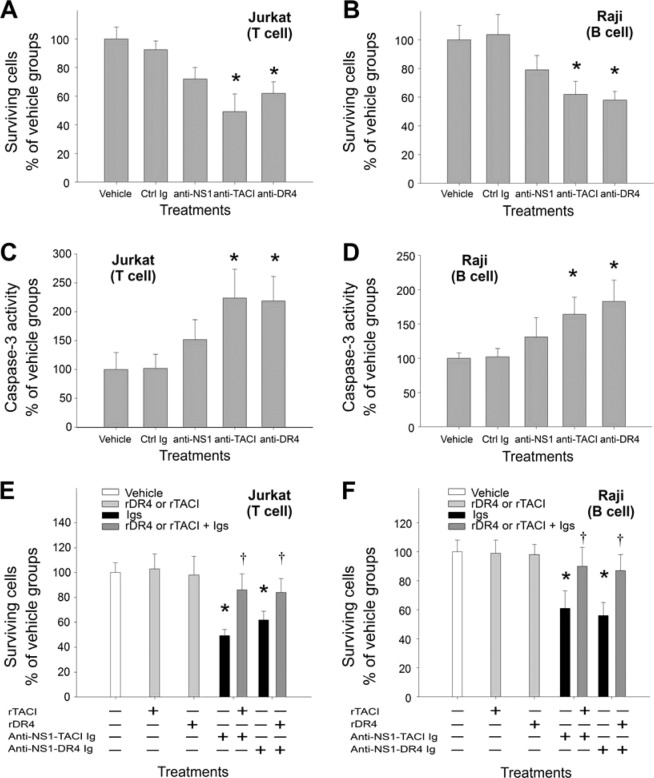
Antibody-induced cell death in immortalized lymphocytic Jurkat (T cell) and Raji (**B** cell) cell lines. The results of Jurkat (**A**) and Raji (**B**) cell survival in response to 72 h preimmune (Ctrl Ig), anti-NS1, anti-TACI, and anti-DR4 Ig treatments are shown. Respective levels of caspase-3 activation were also recorded (**C**,**D**). Recombinant proteins rTACI and rDR4 were used to neutralize the specific antibody effect; the cell survival of Jurkat and Raji cells was also recorded after the treatments (**E**,**F**). *P < 0.05, compared to respective vehicle groups (**C**–**F**). ^†^*P* < 0.05 compared to respective groups without supplements of recombinant protein (**E**,**F**). n = 6, 3 experiments with 2 replicates.

Cell death was examined *in vitro* to determine whether the survival of B cells and T cells is influenced by anti-NS1, anti-TACI, and anti-DR4 Igs. Analytical data showed that 72-h treatment with rabbit anti-TACI and anti-DR4 Igs induced considerable cell death in immortalized B (Raji) and T (Jurkat) cell lines (Fig. 2A,B). Treatments with rabbit anti-TACI and anti-DR4 Igs also increased the sub-G1 population (Suppl. Fig. 1) and the activation of caspase-3 (Fig. 2C,D). Although using an equal dose as anti-TACI and anti-DR4 Igs, the anti-NS1 Ig treatments did not induce significant cell death in Raji and Jurkat cells, the anti-NS1 Ig-treated group still tended to exhibit high cell death and caspase-3 activity levels (Fig. 2A–D and Suppl. Fig. 1).

To examine whether anti-death receptor Ig fractions (Fig. 1B) of polyclonal anti-NS1 Igs induce cell death of lymphoid cell lines, we used affinity-enriched fractions of the anti-NS1 Ig (anti-NS1-TACI Ig and anti-NS1-DR4 Ig) plus additional proteins [e.g. recombinant glutathione S-transferase (rGST; a negative control protein^23,30,31^), recombinant NS1 (rNS1), recombinant TACI (rTACI) and recombinant DR4 (rDR4)] to perform neutralization experiments. Data indicated that anti-NS1-DR4 and anti-NS1-TACI Ig treatments induced cell death of Raji and Jurkat cells in a dose dependent manner (Suppl. Fig. 2); and treatments of rNS1 but not rGST neutralized cell-death inducing property of anti-NS1-DR4 Igs (Suppl. Fig. 3A,B). Additionally, anti-NS1-DR4 Ig fractions prepared from animals immunized with single serotype (DENV-2 NS1 alone) and heterologous serotype (DENV-2 NS1 + DENV-3 NS1), exhibited similar levels of cell-death inducting property (Suppl. Fig. 3C,D). This suggests that the experimental system is feasible, and the autoantibody production of NS1 immunized animals is not influenced by immunizations of single or heterologous serotype NS1. We found that treatments with rTACI and rDR4 considerably reduced anti-NS1-TACI and anti-NS1-DR4 Ig-induced cell death in both Raji and Jurkat cells (Fig. 2E,F, rDR4 or rTACI + Igs group). This suggests that the anti-TACI and anti-DR4 subfractions of anti-NS1 Igs are still able to induce lymphocytic cell death if the titer is high enough.

### Elicitation of anti-TACI and anti-DR4 antibodies caused abnormal B cell development in mouse bone marrow

**Figure 3:**
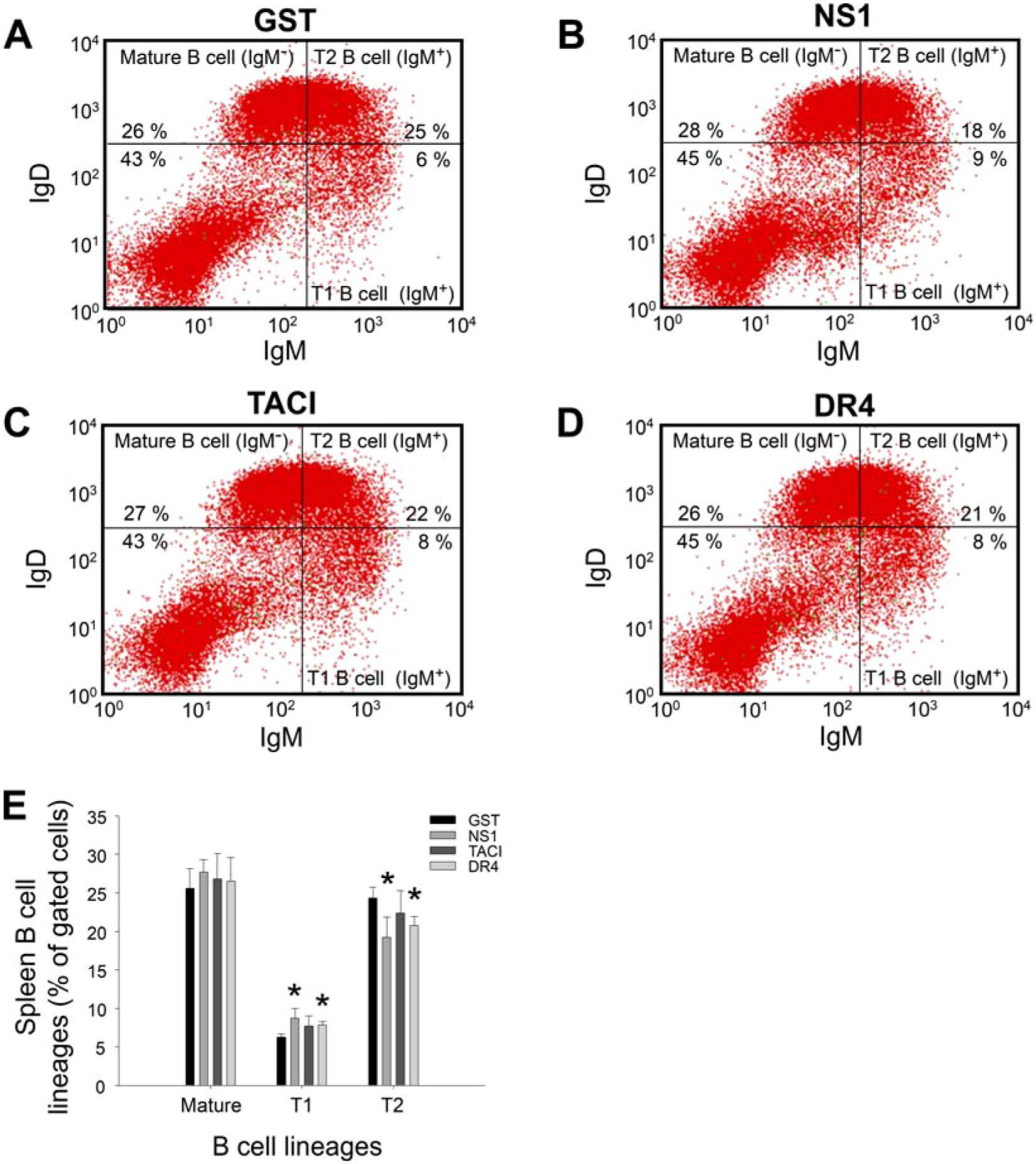
Mouse splenic follicular and transitional T1, and T2 B cell subsets after immunizations with GST, NS1, TACI and DR4 recombinant proteins. Flow cytometry analysis of splenic B cell precursors using IgD and IgM markers was performed after mice were immunized with GST (**A**), NS1 (**B**), TACI (**C**) and DR4 (**D**) recombinant proteins. Quantification analyses revealed that mature (IgD^+^IgM^-^) and transitional T1 (IgD^-^IgM^+^) and T2 (IgD^+^IgM^+^) B cell populations in the spleen were not considerably changed after immunizations with GST, NS1, TACI and DR4 recombinant proteins (**E**). n = 6, 3 independent experiments with 2 replicates.

To analyze whether anti-TACI and anti-DR4 antibody fractions cause an abnormal lymphocytic response *in vivo*, we first analyzed the T cell and B cell populations in C57BL/6J mice immunized with rGST, rNS1, rTACI, and rDR4 for 2 immunization cycles. Because DHF is considered to be associated with secondary DENV infection^2^, 2 immunization cycles of these proteins, particularly NS1, were performed. We found that anti-GST, anti-NS1, anti-TACI, and anti-DR4 Ig titers were markedly elicited in the experimental mice after 2 immunization cycles (Suppl. Fig. 4). Following previously described methods^32^, we found that splenic CD3^+^CD4^−^CD8^−^, CD3^+^CD4^+^CD8^−^, and CD3^+^CD4^−^CD8^+^ T cell populations were not affected after 2 immunization cycles of rGST, rNS1, rTACI, and rDR4 (Suppl. Fig. 5). We analyzed the B cell markers IgM and IgD to detect mature (IgD^+^IgM^−^) and transitional (T1: IgD^−^IgM^+^ and T2: IgD^+^IgM^+^) B cells and the markers CD21/CD35 and CD23 to identify follicular (CD23^+^CD21/CD35^int^) and marginal zone B cells (CD23^−^CD21/CD35^hi^). We also analyzed splenic B cells according to previous reported methods^33,34^. Similar to the results of T cell analysis, considerable changes were not observed in some splenic B cell populations, including follicular (Fo: CD23^+^CD21/CD35^int^) and marginal zone (MZ: CD23^−^CD21/CD35^hi^) B cells (Suppl. Fig. 6), in the mice after immunization with the recombinant proteins. However, at an earlier developmental stage (Suppl. Fig. 7), considerable changes were observed in transitional (T1: IgD^−^IgM^+^ and T2: IgD^+^IgM^+^) B cells after immunization with rNS1 and rDR4 (Fig. 3, GST vs. NS1 and DR4 groups). Although not obvious, the TACI group tended to exhibit similar changes (Fig. 3). Because the T1 populations were increased and the T2 populations were suppressed (Fig. 3, NS1 and DR4 groups), and according to the B cell differentiation process (Suppl. Fig. 7), anti-NS1 and anti-DR4 antibodies may block the transition from T1 to T2 cells. This explanation is consistent with findings of previous studies indicating that primary B cells express TRAIL receptors^35–37^, and transitional T2, but not T1, B cells express TACI^38,39^.

**Figure 4:**
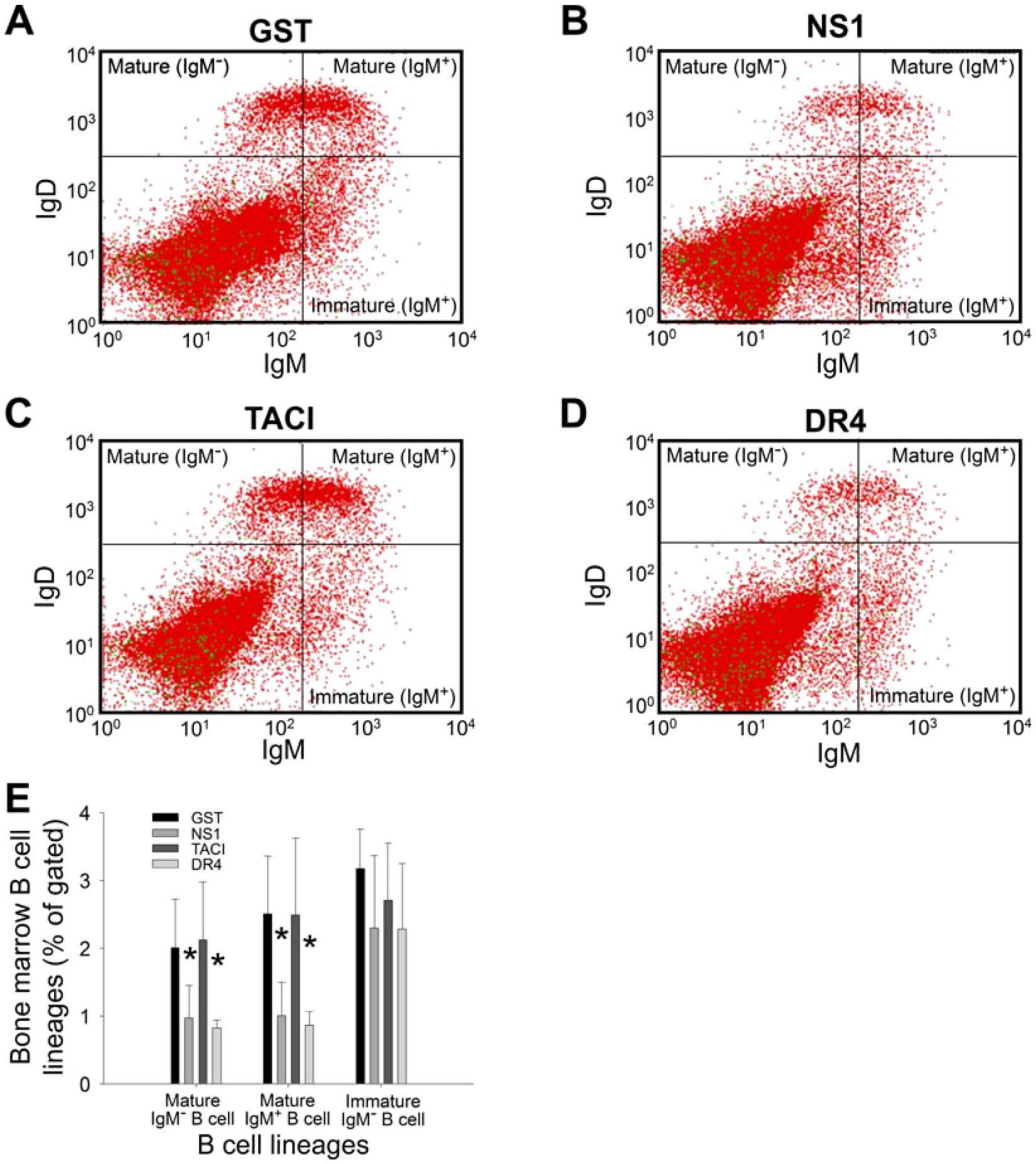
Bone marrow IgM^+^ B cell precursors were suppressed after rNS1 and rDR4 immunizations. Flow cytometry analysis of bone marrow B cell subsets using IgD and IgM markers was performed after mice were immunized with GST (**A**), NS1 (**B**), TACI (**C**) and DR4 (**D**) recombinant proteins. Quantification analyses revealed that mature B cell populations (IgD^+^IgM^+^, IgD^+^IgM^+^) (B220 gated; B220^+^) but not immature B cells (IgD^-^IgM^+^) (B220^+^) in the bone marrow were significantly suppressed after NS1 and DR4 immunizations but not after GST and TACI immunizations (**E**). *P < 0.05 compared to respective GST groups. n = 6, 3 independent experiments with 2 replicates.

**Figure 5:**
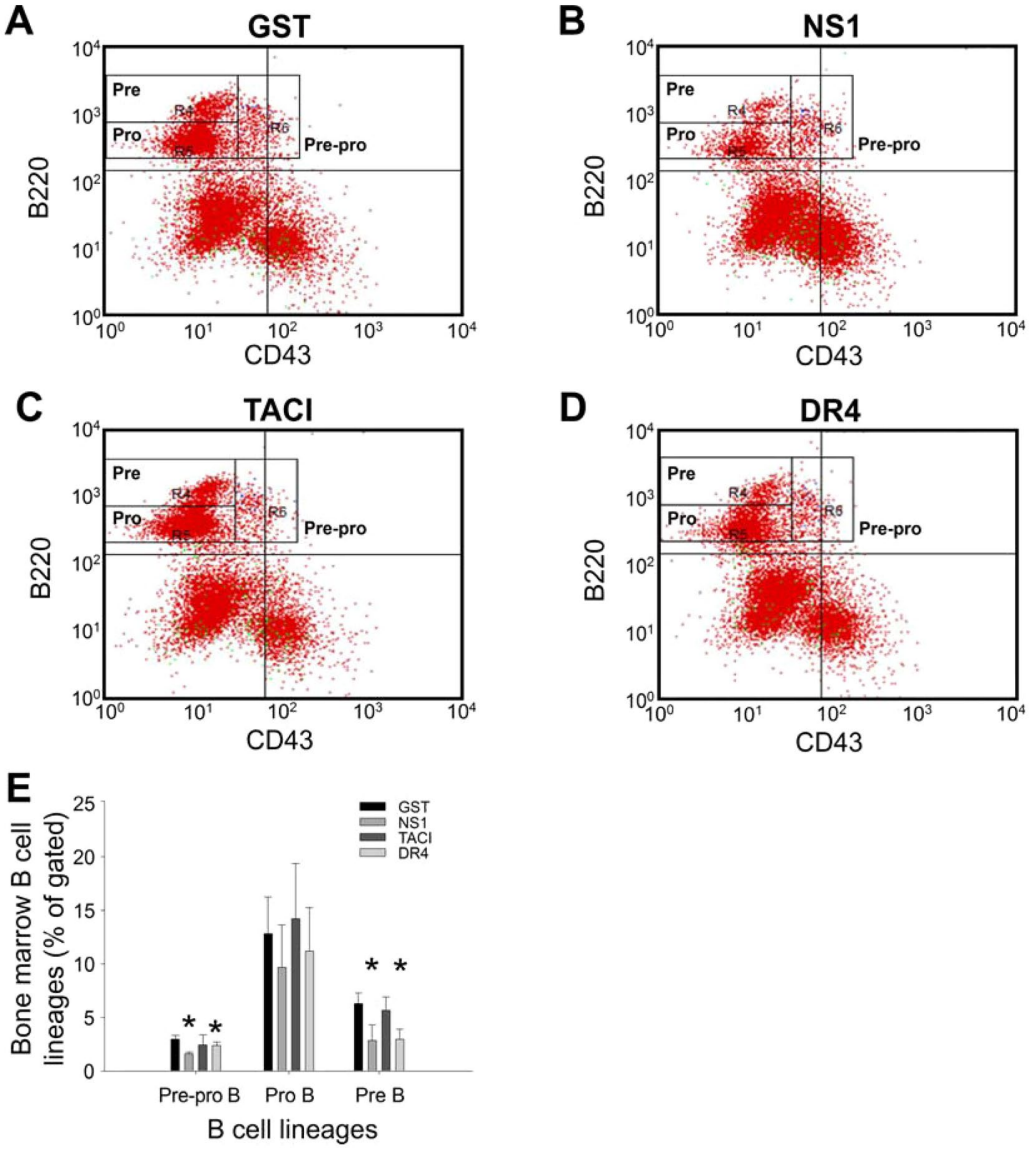
Bone marrow B220^+^CD43^+^ pre-pro-B and B220^+^CD43^-^ pre-B cells were suppressed after rNS1 and rDR4 immunizations. Flow cytometry analysis of bone marrow B cell precursors using B220 and CD43 markers was performed after mice were immunized with GST (**A**), NS1 (**B**), TACI (**C**) and DR4 (**D**) recombinant proteins. Quantification analyses revealed that pre-pro-B (B220^+^CD43^+^) pre-B (B220^+^CD43^-^) cell populations in the bone marrow were significantly suppressed after NS1 and DR4 immunizations but not after GST and TACI immunizations (**E**). *P < 0.05 compared to respective GST groups. n = 6, 3 independent experiments with 2 replicates.

To investigate whether immunization with the recombinant proteins influences different stages of B cell development, we analyzed bone marrow B220^+^ B cells using the B cell markers IgM and IgD to detect mature (IgD^+^IgM^−^, IgD^+^IgM^+^) and immature (IgD^−^IgM^+^) B cells and the markers B220 and CD43 to identify pre-pro-B (CD43^+^B220^+^), pre-B, and pro-B (CD43^−^B220^+^) cells^40–43^. Notably, we found that mature (IgD^+^IgM^−^, IgD^+^IgM^+^) and immature (IgD^−^IgM^+^) B cells in the mouse bone marrow were considerably decreased after 2 immunization cycles of rNS1 and rDR4, but not rGST and rTACI (Fig. 4, *P < 0.05, vs. respective GST groups). Consistently, analyses of fluorescence labeling of the B cell markers B220 and CD43 revealed that pre-pro-B (CD43^+^B220^+^) and pre-B (CD43^−^B220^+^, the population in upper gated area) cells also were markedly decreased after immunization with rNS1 and rDR4 (Fig. 5, *P < 0.05, vs. respective GST groups). Moreover, immunization with NS1 elicited anti-DR4 Ig titers (Fig. 1B). These results (Figs. 4 and 5) suggest that the elicitation of anti-DR4 Ig titers is associated with the suppression of bone marrow B cell populations.

### Preexisting anti-DR4 antibodies reduced production of neutralizing antibody in mice

**Figure 6:**
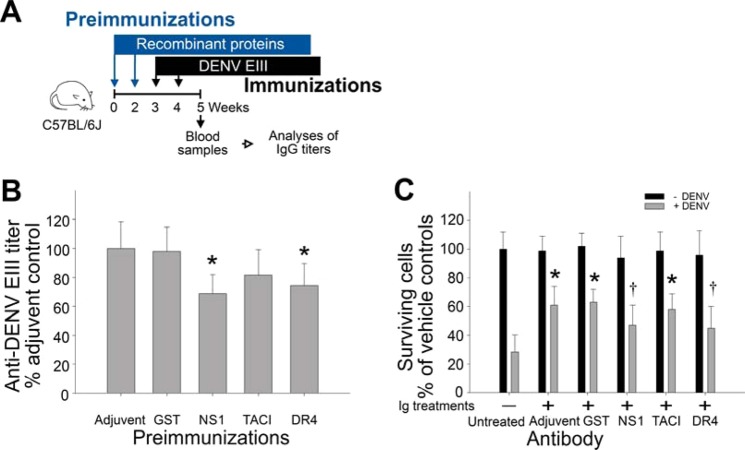
Preimmunization of rNS1 and rDR4 suppressed elicitation of neutralizing anti-DENV EIII titers. Experimental mice were firstly immunized with adjuvant (without mixed with proteins), rGST, rNS1, rTACI and rDR4 for 2 cycles, and then immunization with rEIII for additional 2 cycles (**A**) (experiment outline). The anti-EIII Ig titers were analyzed using ELISA in the 5^th^ week (**B**). The neutralizing properties of these anti-EIII Ig were analyzed by protection of BHK-21 cells against DENV infection *in vitro*. The cell survival rates were recorded after 96 h challenges of DENV (MOI = 0.5) and 96 h treatments of heat-inactivated anti-serum (20 µL/well of microtiter plate) from respective groups (**C**). *P < 0.05 compared to respective GST groups (**B**), compared to untreated groups (**C**). ^†^*P* < 0.05 compared to GST groups (**C**). n = 12, 3 independent experiments with 4 replicates. The mouse drawing used in this figure was originally published in the Blood journal: Huang, H. S., Sun, D. S., Lien, T. S. and Chang, H. H. Dendritic cells modulate platelet activity in IVIg-mediated amelioration of ITP in mice. Blood, 2010; 116: 5002–500958^89^; © the American Society of Hematology.

The elicitation of anti-DR4 Ig titers is associated with development of abnormal B cell subsets in the mouse spleen and bone marrow (Figs. 3–5). Although the total circulating IgM and IgG levels were not changed (Suppl. Fig. 8), whether such B cell abnormalities are sufficient to result in suppressed humoral immunity remains unclear. Thus, we performed functional analysis to investigate whether such B cell abnormalities lead to immunosuppression. DENV envelope protein domain III (EIII) contains glycosaminoglycan property; by which DENV utilizes EIII to bind to cell surface receptors and enter target cells^44–46^. Thus, anti-DENV EIII neutralizing antibodies have a protective effect against DENV infection^47,48^. Following the experimental methods in the previous section, we first immunized mice with recombinant proteins, rGST, rNS1, rTACI, and rDR4, for 2 immunization cycles and then immunized all groups of mice with recombinant EIII (rEIII) for 2 additional cycles (Fig. 6A, experimental outline). We hypothesized that if anti-NS1 and anti-DR4 Ig-induced B lymphocyte defects are sufficient to result in suppressed humoral immunity, we would observe a lower anti-EIII antibody titer. Consistent with our hypothesis, a relatively lower anti-EIII Ig titer was elicited in mice preimmunized with rNS1 and rDR4 (Fig. 6B, *P < 0.05, vs. adjuvant and GST groups). To analyze whether such anti-EIII Igs also display different protective properties against DENV infection, we used anti-EIII Igs from different mouse groups to protect BHK-21 cells from DENV infection *in vitro*. Notably, the anti-EIII sera from adjuvant, rGST, and rTACI-preimmunized mice displayed considerable protective effects against DENV infection compared with the untreated control groups (Fig. 6C, adjuvant, GST, and TACI groups, *P < 0.05). However, the anti-EIII sera from rNS1 and rDR4-preimmunized mice did not display significant protective effect (Fig. 6C, NS1 and DR4 groups). The coefficient of determination (R-squared; R^2^) analysis revealed that the anti-EIII IgG levels are indeed positively correlated with protected cell viability of DENV-infected cells (Suppl. Fig. 9). These results collectively suggest that the elicitation of DR4 cross-reactive Igs causes the abnormal development of bone marrow B cells and results in suppressed humoral immunity in mice.

## Discussion

Preexisting nonneutralizing enhancing antibodies are crucial for inducing the ADE phenomenon in DENV infections^49^. It is unclear how these nonneutralizing Igs are elicited before the ADE phenomenon. Recent findings suggest that even the neutralizing Igs at a dose below the neutralizing concentration exhibit the enhancing properties^48,50^. Anti-DENV EIII monoclonal antibodies (mAbs) derived from immortalized memory B cells of patients after primary or secondary infections were either serotype specific or cross-reactive and potently neutralized DENV infection^48^. However, a notable finding is that all these mAbs enhanced infection at subneutralizing concentrations^48^. This evidence suggests that even if the Ig concentration is not sufficiently high, the neutralizing antibodies produced by B cells may still induce the pathogenic ADE effect. In this study, we found that the preexisting anti-NS1 and anti-DR4 Igs could suppress the neutralizing antibody titers in the mice subsequently immunized with DENV EIII (Fig. 6). This finding provides a possible explanation for the production of such nonneutralizing or subneutralizing antibodies before ADE.

Another puzzling aspect of humoral immunity suppression after DENV infection is the delayed induction of anti-DENV Igs during secondary DENV infection^51^. One valuable observation from DENV-endemic regions is that the prevalence of the anti-DENV antibody in the general population can reach up to 90%^51–53^. This observation suggests that the majority of the population in endemic regions has already been infected with DENV at least once. In cases of infection with ordinary pathogens, a rapid anamnestic antibody response should occur within 1–3 days after the same antigens are reencountered^51,54^. However, the anti-DENV Igs display late induction that peaks at 6–10 days after fever onset during secondary infection in DHF patients^10,51^. The relevant mechanism is unclear. According to the original antigenic sin hypothesis, a weak immune response occurs during secondary DENV infections^13,14^. However, the mechanism on the elicitation of insufficient humoral immunity in dengue remains elusive. In this study, we found that preexisting anti-NS1 and anti-DR4 antibody fractions could suppress B cell development (Figs. 3–5) and the subsequently elicited neutralizing antibodies against DENV (Fig. 6). In addition, our results revealed that the anti-DR4 Ig affects immature B cell precursors (Figs. 3 and 5). The suppression of newly produced B cells in the secondary DENV-infected hosts may result in preferred antibody production from those mature B cell clones been developed during the primary infections. Thus, compare to the original antigenic sin hypothesis, the suppressive effect of DENV-elicited anti-death receptor Igs on the antibody production by B cells is an alternative explanation for the phenotype in biased and suppressed humoral immunity, as well as the delayed and weak induction of anti-DENV antibodies during secondary infection (Suppl. Fig. 10).

In this study, we demonstrated that DENV NS1 can elicit anti-death receptor Ig fractions. We also demonstrated that the elicitation of the anti-DR4 fraction is associated with abnormal populations of bone marrow and splenic B cell precursors and suppressed humoral immunity. Our data revealed that, although the elicitation of anti-TACI Ig was not statistically significant compared with the elicitation of anti-DR4 Ig fractions, anti-TACI Igs still tended to exert a suppressive effect under certain conditions (e.g., in Figs. 2, 5, and 6B). Through interaction with TACI, BCMA, and BAFFR on B cells, BAFF enhances B cell differentiation and activation^38,55^. BAFF overexpression is associated with autoantibody production in autoimmune diseases^56^. BAFF promotes autoantibody production through TACI-dependent activation of transitional B cells^57^. TACI plays a critical role in the regulation of B cell differentiation and activation^38,58,59^. TACI^−/−^ mice exhibited accumulation and activation of B cells^60^, and TACI deficiency leads to an autoimmune-prone phenotype^59^. By contrast, TACI-Fc leads to decreased B cell numbers and potently blocks humoral immune responses^61–63^. Furthermore, the induction of TACI expression levels enhances B cell apoptosis *in vivo*^64^. Similar to TACI deficiencies, deficiencies in TRAIL receptors lead to an inflammation-prone phenotype^65,66^. TRAIL^−/−^ mice are more susceptible to arthritis and diabetes^67^. Moreover, recombinant TRAIL was demonstrated to inhibit autoimmune diseases in numerous animal models, such as those of rheumatoid arthritis, autoimmune encephalomyelitis, and autoimmune thyroiditis^68,69^. TRAIL sensitizes B cells to apoptosis and induces B cell apoptosis^35–37^. By cross-linking with the cell surface receptors, anti-DR4 Igs induce similar signaling to that of the native ligand TRAIL^70–72^. These findings collectively suggest that the both TACI and DR4 pathways play negative roles in the regulation of immune homeostasis and may explain why the elicitation of anti-NS1 and anti-DR4 Igs suppressed humeral immunity *in vivo*. Because NS1 immunization elicited both anti-DR4 and anti-TACI Ig fractions in this study (Fig. 2B), a synergy between these 2 antibody fractions may be theoretically possible and worth further investigation.

DENV-elicited pre-existing antibodies have been hypothesized to affect secondary DENV infection in many ways. For example, antibody dependent enhancement (ADE) of viral uptake by FcR-bearing cells and productive infection^9–11^, anti-platelet and anti-endothelial cell autoantibodies associated thrombocytopenia and vascular leakage^15–17,19–26^. To summarize and integrate the results described in this report with these previous findings, we postulate a hypothetical model following the time line of DHF pathogenesis (Suppl. Fig. 11). Based on the 2-hit model, the levels of anti-platelet and anti-endothelial cell autoantibody fractions (second-hit) are not sufficient to elicit hemorrhage pathogenesis, only when the DENV-induced defects (first-hit) are co-occurring^22^. Accordingly, elevated levels of autoantibodies suppress the B cell development, could thereby reduce the anti-DENV-neutralizing Ig production, and lead to stronger DENV (first hit)-induced damages and thus result in an increased risk on the development of DHF (Suppl. Fig. 11). Because the anti-DENV Igs are cross-reactive, virus-Ig complexes subsequently induce antibody dependent enhancement (ADE)-related pathogenesis to further exacerbate the disease (Suppl. Fig. 10).

**Figure 7:**
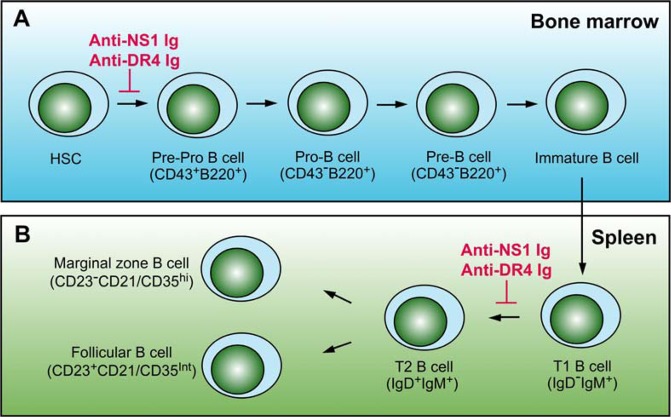
A hypothetical model of anti-NS1 Ig-mediated suppression on mouse B cell. Illustration shows different steps and surface markers of B cell development from hematopoietic stem cells (HSCs) in the bone marrow (**A**) to marginal zone and follicular B cells in the spleen (**B**). After immunization cycles of recombinant NS1 and DR4 proteins, we found that mouse bone marrow B220^+^CD43^+^ pre-pro-B cells (**A**) and splenic IgD^+^IgM^+^ transitional T2 B cell subsets (**B**) were suppressed (red labels), suggesting that the elicitations of anti-NS1 and anti-DR4 Igs can interfere with B cell differentiation.

In summary, we report that the elicitation of anti-death receptor antibody fractions through immunization with rNS1 and rDR4 suppresses B cell development (Fig. 7) and DENV-neutralizing antibody production. This finding may explain the induction of suppressed humeral immunity during DENV infections. In addition, this concept may be useful for developing an effective and safe DENV vaccine. Because human and mouse behave differently in response to DENV infection, more clinical studies are needed to clarify the exact response and regulation in patients with severe dengue.

## Materials and Methods

### DENV, recombinant proteins and antibodies

The DENV-2 (strain PL046) and soluble recombinant proteins DENV NS1 (rNS1; DENV-2 PL046) were obtained and purified using previously described methods^22,24,30^. Recombinant human TNF receptor-ectodomain-IgG Fc fusion proteins, such as TRAIL-R1 (DR4)-IgG Fc (rDR4-Fc), TRAIL-R2 (DR5)-IgG Fc (rDR5-Fc), TRAIL-R3 (DcR1)-IgG Fc (rDcR1-Fc), TRAIL-R4 (DcR2)-IgG Fc (rDcR2-Fc), BCMA-IgG Fc (rBCMA-Fc) and BAFFR-IgG Fc (rBAFFR-Fc), were purchased from R&D Systems (Minneapolis, MN). Recombinant DENV envelope protein domain III (EIII) was purchased from ProSpec (East Brunswick, NJ). Recombinant TACI ectodomain (rTACI) was expressed by *Escherichia coli*. Preimmune Ig and anti-NS1 Ig from New Zealand white rabbits (*Oryctolagus cuniculus*) were obtained before and after immunizations of NS1 recombinant proteins^24,30^. The anti-DR4 Ig was prepared from recombinant DR4-immunized rabbits as described^23^. The anti-TACI Ig was prepared from rTACI-immunized rabbits. All serum samples of protein immunized rabbits were collected 7–10 days after the 5^th^ immunization cycle of aforementioned recombinant proteins. To enhance the immunization efficiency in rabbit and mouse experiments, the recombinant proteins (250 μg/rabbit; 50 μg/mouse) were emulsified with complete Freund’s adjuvant (Sigma-Aldrich, St. Louis, MO) in the first immunization; in the later boosting cycles, incomplete Freund’s adjuvant (Sigma-Aldrich) was used 1:1 (v/v) mixed with recombinant proteins, 100 μg/rabbit; 25 μg/mouse) as described^30,73,74^. The induction of IgG titers against specific antigens was confirmed using enzyme-linked immunosorbent assay (ELISA). A batch of 8 rabbits was immunized with NS1, in which anti-sera from 3 rabbits with relatively higher anti-DR4 and anti-TACI IgG titers were used. The IgG fractions were obtained and purified from rabbit serum samples using a protein-A column connected to a peristaltic pump (GE Healthcare Life Sciences, Little Chalfont, UK) that operated at a flow rate of 0.5–1 mL min^−1^. As described^23^, anti-NS1-DR4 and anti-NS1-TACI Igs were further prepared from protein A-fractions of anti-NS1 Ig through affinity purifications using rDR4- and rTACI-conjugated beads, respectively. Using SDS-PAGE and NIH-Image J software (version 1.6.2), the purified Igs were estimated approximately 87%–91% pure. Experimental mice and rabbits were maintained at a specific pathogen free condition with consistent temperature and humidity controls, and 12 h light/dark cycles^75–77^. All protocols used in rabbit and mouse experiments were carried out in accordance with the guidelines of and approved by the Institutional Animal Care and Use Committee of Tzu-Chi University (approval ID: 101019).

### Patient samples

Using the guidelines reported by the WHO^78,79^, dengue cases (21 with DF; 14 with DHF) were classified, and their serum samples were collected as described^23^. The DF cases were classified by manifestations of fever accompanied by at least 2 of the following symptoms: severe headache, retro-orbital pain, myalgia, arthralgia, and a rash. The DHF cases were also defined according these clinical manifestations as well as additional hemorrhagic manifestations, including positive tourniquet test results, hemoconcentration, or additional signs of plasma leakage^78,79^. Following previously described methods^24,30^, serum samples were collected from patients and tested using at least 2 of the following 4 methods: (1) virus infection and isolation from mosquito C6/36 cells, (2) serotype-specific RT-PCR, (3) DENV-specific IgM or IgG measurement, and (4) a hemagglutination inhibition test^23^. The serum samples were collected 3–7 d after the onset of fever. DENV-specific IgM and IgG antibodies were analyzed using anti-dengue IgM and IgG capture ELISA kits (PanBio, Brisbane, Australia) according to the manufacturer’s instructions. The personal information of all patients and donors was de-identified from the samples and only aggregated data was used for data analysis. All protocols in this study concerning the human subjects were conducted in accordance with the guidelines of, and were approved by the Institutional Review Board (approval ID: E6A0021538-02). Informed consent was obtained from all participants and/or their legal guardian/s.

### ELISA analysis

A standard ELISA protocol was used to determine the antibody titers from patients and rabbits against the rGST, rNS1, rDR4 and rTACI. Recombinant fusion proteins including DR4-Fc, BCMA-Fc and TACI-Fc (40 µg/mL; 40 µL per well) were coated on microtiter plates as binding substrates. After incubation with the patients’ sera (1:100 diluted) or purified rabbit Igs (0.1 µg/100 µL/well), the levels of the substrate-bound Igs were probed using specific horseradish peroxidase-conjugated antibodies, and were then measured as described previously^24,30^.

### Cell lines, cell viability and caspase activity analyses

Jurkat (ATCC® TIB152™), an immortalized line of human T lymphocytes, and Raji (ATCC® CCL86™), an immortalized line of human B lymphocytes, were maintained in RPMI cell culture medium, supplemented with 10% fetal bovine serum (FBS), 2 mM glutamine, 100 units/mL penicillin, 100 µg/mL streptomycin, based on standard cell culture protocols^80,81^. The cell viability of these 2 cell lines was analyzed using a WST-1 kit (Roche Life Science, Penzberg, Germany)^30,82,83^. The tetrazolium salt WST-1 is cleaved to a soluble formazan by the succinate-tetrazolium reductase system, which is only active in metabolically intact cells, and the formazan is measurable at 450 nm using an ELISA reader (Molecular Devices, Sunnyvale, CA)^84^. The surviving Jurkat and Raji cells were measured after treatments with varying antibodies (100 μg/mL) for 72 h. The 72 h groups of Ig treated cells were also applied to caspase-3 analysis, which were conducted using caspase-activity assay kits purchased from R&D systems (Minneapolis, MN, USA). Baby hamster kidney BHK-21 cells (ATCC® CCL-10)^85^ were maintained in DMEM 10% FBS, 4.5 g/L glucose, 6 mM glutamine cell culture medium and were used in the analysis of anti-envelop EIII protein neutralizing antibody. The serum samples from mice immunized with 2 cycles of recombinant GST, NS1, TACI and DR4, and 2 additional cycles with recombinant DENV EIII were collected and placed in a 56 °C water bath for 20 m to inactivate the complement. The BHK-21 cells were cultured in 96 well dishes in serum free medium, with aforementioned antisera supplements (20 µL/well of microtiter plate). The cell survival was then analyzed after challenged with or without DENV (MOI = 0.5) for 96 h using a WST-1 kit (Roche). The mosquito (*Aedes albopictus*) cell line C6/36 (ATCC® CRL1660™) was maintained using EMEM (EBSS) 10% FBS, 2 mM glutamine at 28 °C, and was used for identification of DENV in patient serum samples.

### Mouse B cell analyses

Wild type C57BL/6J mice aged 8–12 weeks were purchased from the National Laboratory Animal Center (Taipei, Taiwan). All animals were housed in the Tzu-Chi University Animal Center in a specific-pathogen-free, temperature and lighting controlled environment with free access to filtered water and food. The experimental procedures were approved by the Animal Care and Use Committee of Tzu-Chi University (approval ID: 101019). Fluorescence-labeled anti-IgM, anti-IgD, anti-B220, anti-CD43, anti-CD21/CD35 and anti-CD23 Igs that used in the flow cytometry B lymphocyte analyses, were purchased from (eBioscience, San Diego, CA and BD Biosciences, Franklin Lakes, NJ). Anti-IgM and anti-IgD were used to identify subsets as described. Anti-CD21/CD35 and anti-CD23 Igs were used to identify spleen B cell subsets following previously described methods. Following previously described methods^33,34,40–43^, we analyze spleen transitional T1, T2, and follicular and marginal zone, mature B cells, bone marrow mature (IgD^+^IgM^-^, IgD^+^IgM^+^) and immature (IgD^-^IgM^+^) B cells, and pre-pro-B (CD43^+^B220^+^), pre-B and pro-B (CD43^-^B220^+^) cells. A flow cytometer (FACSCalibur; BD Biosciences, San Jose, CA, USA) was used in this study as described^86–88^. C57BL/6J mice were immunized with rGST, rNS1, rDR4 and rTACI for 2 immunization cycles (50 µg immunogen/mouse/immunization cycle) in 1 wk intervals and then the bone marrow and spleen lymphocytes were analyzed 7 d later using aforementioned B cell markers. To analyze the potential suppressive effect of prior immunizations of NS1 on later induction of neutralizing antibody, C57BL/6J mice were first immunized with rGST, rNS1, rDR4 and rTACI by 2 immunization cycles (50 µg immunogen/mouse/immunization cycle) and then immunized with 2 additional immunization cycles of DENV rEIII in 1 wk intervals. The anti-EIII titer and the DENV-neutralizing property of these polyclonal antibody fractions were then analyzed.

### Statistical analyses

The means, standard deviations, and statistics for the quantifiable data were calculated using Microsoft Office Excel 2003, SigmaPlot 10 and SPSS 17. Significance of data was examined by 1-way ANOVA followed by the post-hoc Bonferroni-corrected t test. The probability of type 1 error α = 0.05 was recognized to be the threshold of statistical significance.

## Supplementary information

Supplementalinformation

## Data Availability

The data used to support the findings of this study are available from the corresponding author upon request.
