# Supplementary material for: Suppressed humoral immunity is associated with dengue nonstructural protein NS1-elicited anti-death receptor antibody fractions in mice
Source: Sci Rep. 2020 Apr 14;10:6294. doi: 10.1038/s41598-020-62958-0 (PMC7156414; doi:10.1038/s41598-020-62958-0)
Supplement: Supplementary file 1 — Supplementalinformation [file 41598_2020_62958_MOESM1_ESM.docx]

Supplemental Materials:

Suppressed humoral immunity is associated with dengue nonstructural protein NS1-elicited anti-death receptor antibody fractions in mice

Chung-Lin Tsai ^1†^, Der-Shan Sun ^1†^, Mei-Tzu Su ^1†^, Te-Sheng Lien ^1†^, Yen-Hsu Chen ^2,3,4^, Chun-Yu Lin ^3,5^, Chung-Hao Huang ^3,5^, Chwan-Chuen King ^6^, Chen-Ru Li ^1^, Tai-Hung Chen ^1^, Yu-Hsiang Chiu ^7^, Chun-Chi Lu ^7^, and Hsin-Hou Chang ^1*^

1. Department of Molecular Biology and Human Genetics, Tzu-Chi University, Hualien, Taiwan.

2. Department of Internal Medicine, Kaohsiung Municipal Ta-Tung Hospital, Kaohsiung, Taiwan.

3. School of Medicine, Graduate Institute of Medicine, Sepsis Research Center, Center of Tropical Medicine and Infectious diseases, Kaohsiung Medical University, Kaohsiung, Taiwan.

4. Department of Biological Science and Technology, College of Biological Science and Technology, National Chiao Tung University, HsinChu, Taiwan.

5. Division of Infectious Diseases, Department of Internal Medicine, Kaohsiung Medical University Hospital, Kaohsiung Medical University, Kaohsiung, Taiwan.

6. Institute of Epidemiology and Preventive Medicine, National Taiwan University, Taipei, Taiwan.

7. Division of Rheumatology/Immunology and Allergy, Department of Internal Medicine, Tri-Service General Hospital, National Defense Medical Center, Taipei, Taiwan.

† These authors share equals contribution

***Correspondence:**

Hsin-Hou Chang, PhD.

Department of Molecular Biology and Human Genetics, Tzu-Chi University, Hualien 970, Taiwan, ROC.

Tel: 886-3-8565301 ext 2667. Fax: 886-3-8578386.

E-mail:hhchang@mail.tcu.edu.tw

**Supplemental Figures 1-11.**

**Suppl. Fig. 1**


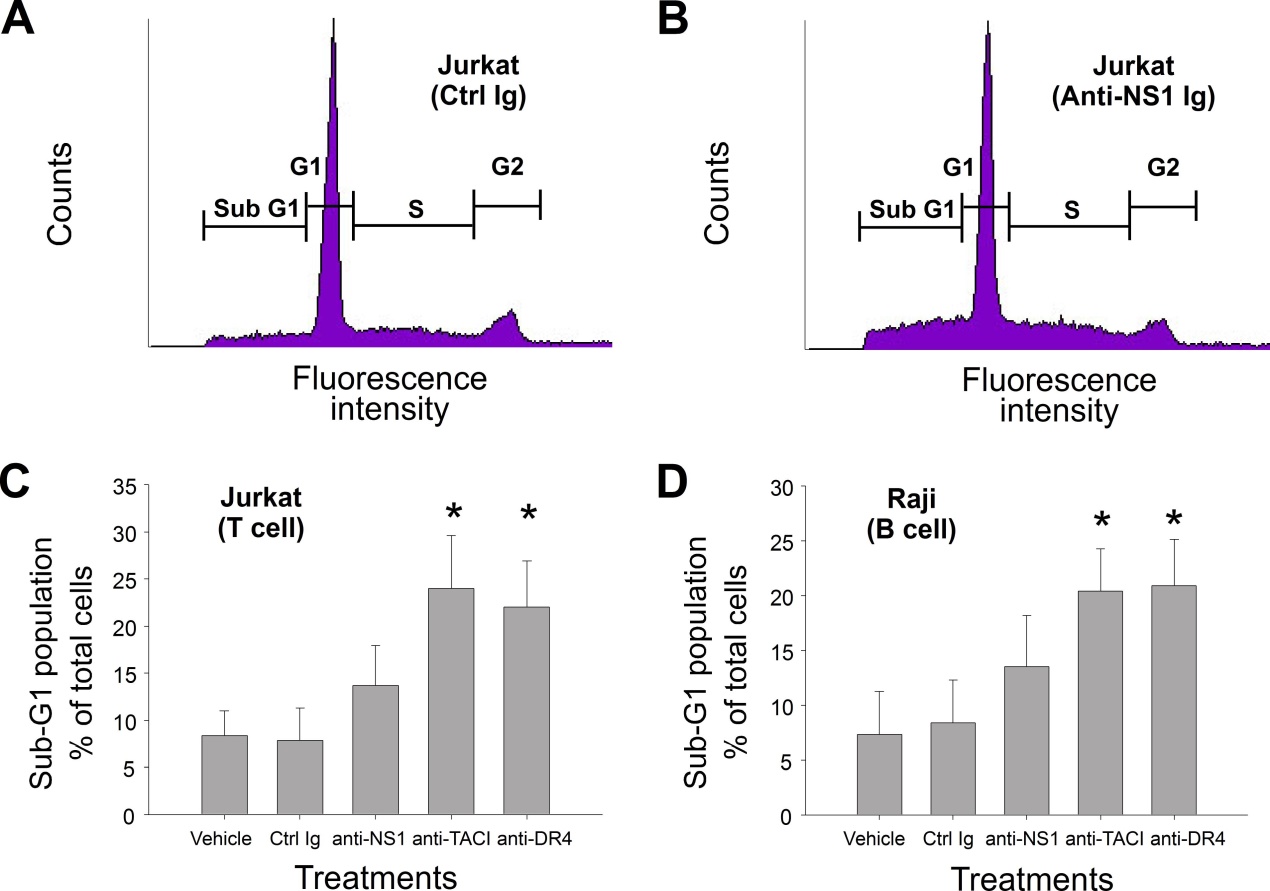


**Suppl. Fig. 1. Antibody-mediated induction of sub-G1 population in immortalized lymphocytic Jurkat (T cell) and Raji (B cell) cell lines.** Flow cytometry analyses of sub-G1 population, an indication of cell death, were indicated (example histograms showed in A and B). Quantification results of Jurkat (C) and Raji (B) cells in response to 72 h preimmune (Ctrl Ig), anti-NS1, anti-TACI, and anti-DR4 Ig treatments are shown. * *P* < 0.05, compared to respective vehicle groups (C and D). n = 6, 3 experiments with 2 replicates.

**Suppl. Fig. 2**


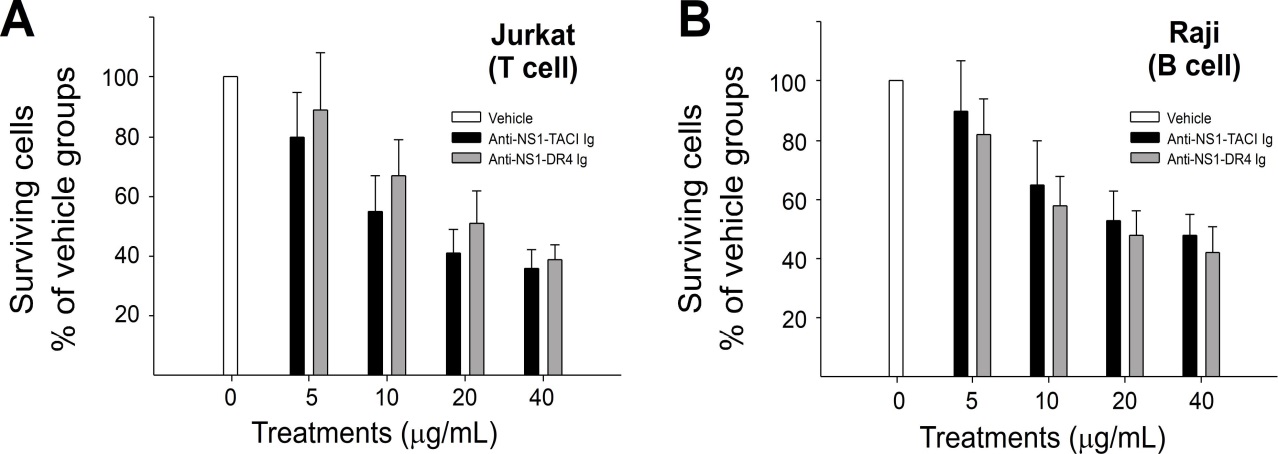


**Suppl. Fig. 2. Dose-dependent antibody-induced cell death in immortalized lymphocytic Jurkat (T cell) and Raji (B cell) cell lines.** The results of Jurkat (A) and Raji (B) cell survival in response to 72 h anti-NS1-TACI and anti-NS1-DR4 Igs treatments are shown. n = 6, 2 experiments with 3 replicates.

**Suppl. Fig. 3**


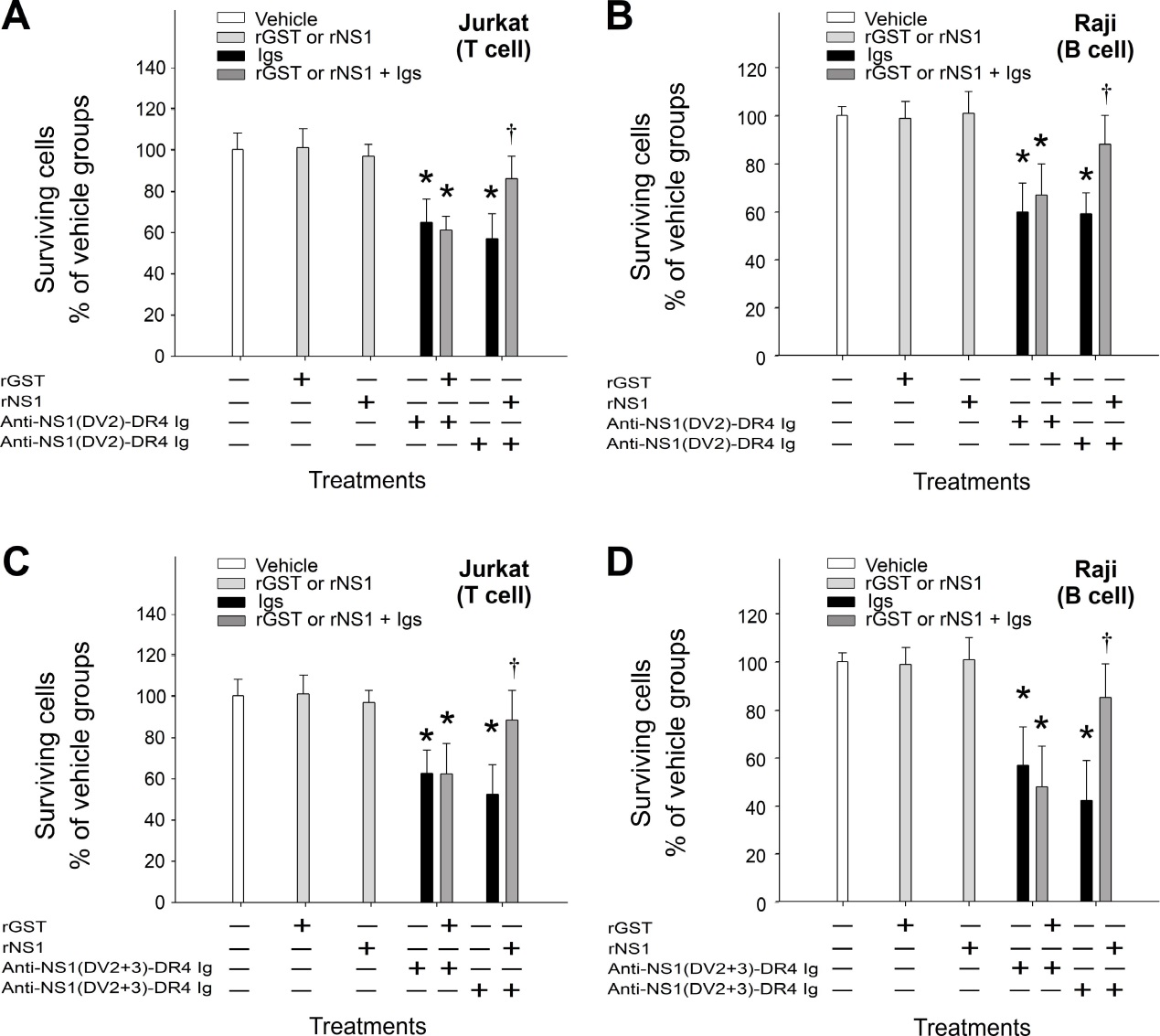


**Suppl. Fig. 3. Recombinant NS1 neutralized autoantibody-induced cell death in immortalized lymphocytic Jurkat (T cell) and Raji (B cell) cell lines.** The results of Jurkat (A, C) and Raji (B, D) cell survival in response to 72 h recombinant proteins and Igs treatments are shown. Recombinant proteins rGST (negative control) and rNS1 were used to neutralize the specific antibody effect; the cell survival of Jurkat and Raji cells was also recorded after the treatments. Anti-NS1(DV2)-TACI Igs are prepared from anti-NS1 Ig fractions of animals with single serotype DENV-2 NS1 immunizations (2 cycles). By contrast, anti-NS1(DV2+3)-TACI Igs are prepared from anti-NS1 Ig fractions of animals with multiple serotype DENV-2 NS1 and DENV-3 NS1 immunizations (2 cycles; 1 cycle of DENV-2 NS1 + 1 cycle of DENV-3 NS1). * *P* < 0.05, compared to respective vehicle groups; ^†^ *P* < 0.05 compared to respective groups without supplements of recombinant protein. n = 6, 3 experiments with 2 replicates.

**Suppl. Fig. 4**


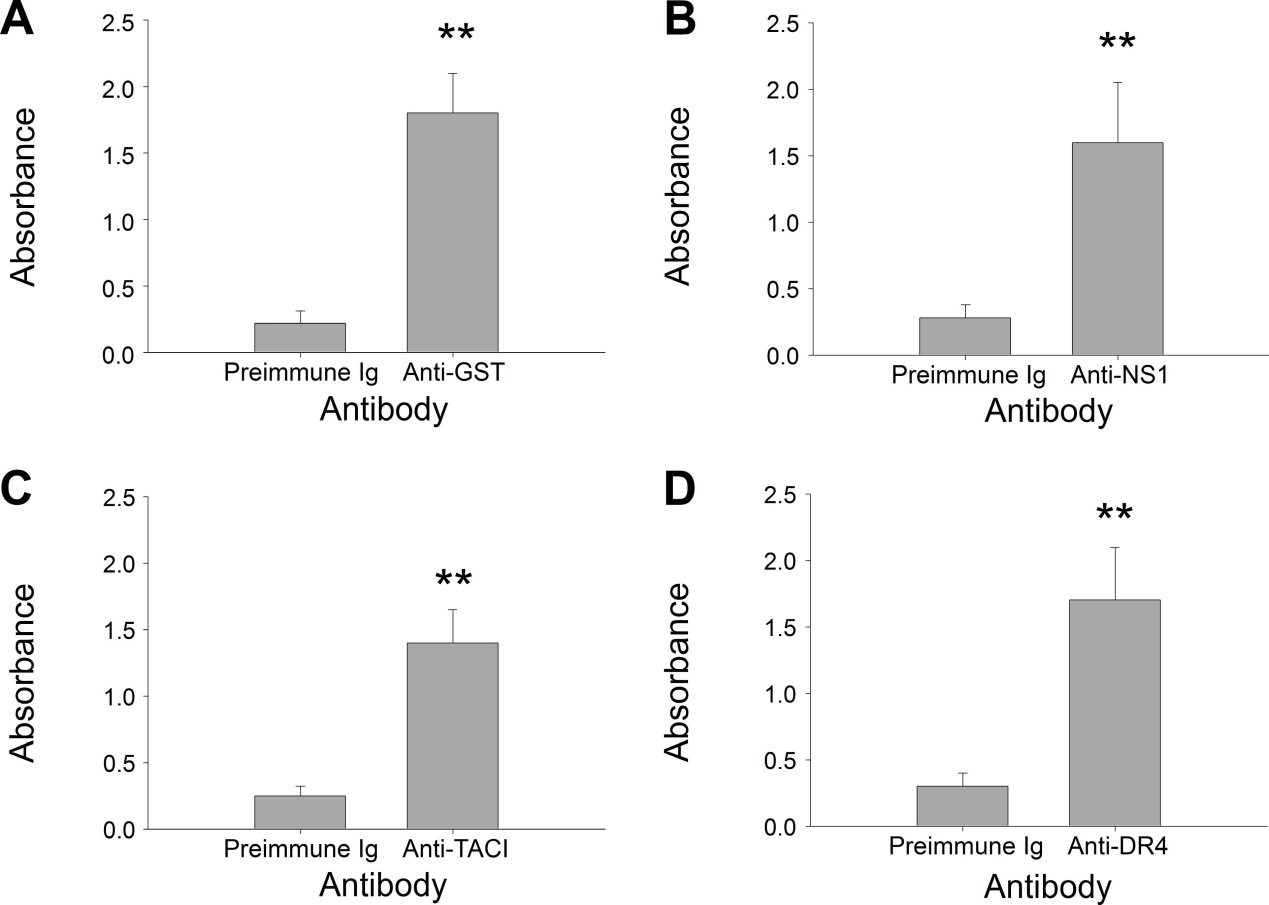


**Suppl. Fig. 4. Elicitation of antibody titers in GST, NS1, TACI and DR4 recombinant proteins immunized mice.** Antibody titers against GST (A), NS1 (B), TACI (C) and DR4 (D) of experimental mice before (preimmune Ig groups) and after 2-cycle immunizations with rGST, rNS1, rTACI and rDR4, were analyzed using ELISA, respectively. ** *P* < 0.01; n = 6, 3 independent experiments with 2 replicates.

**Suppl. Fig. 5**


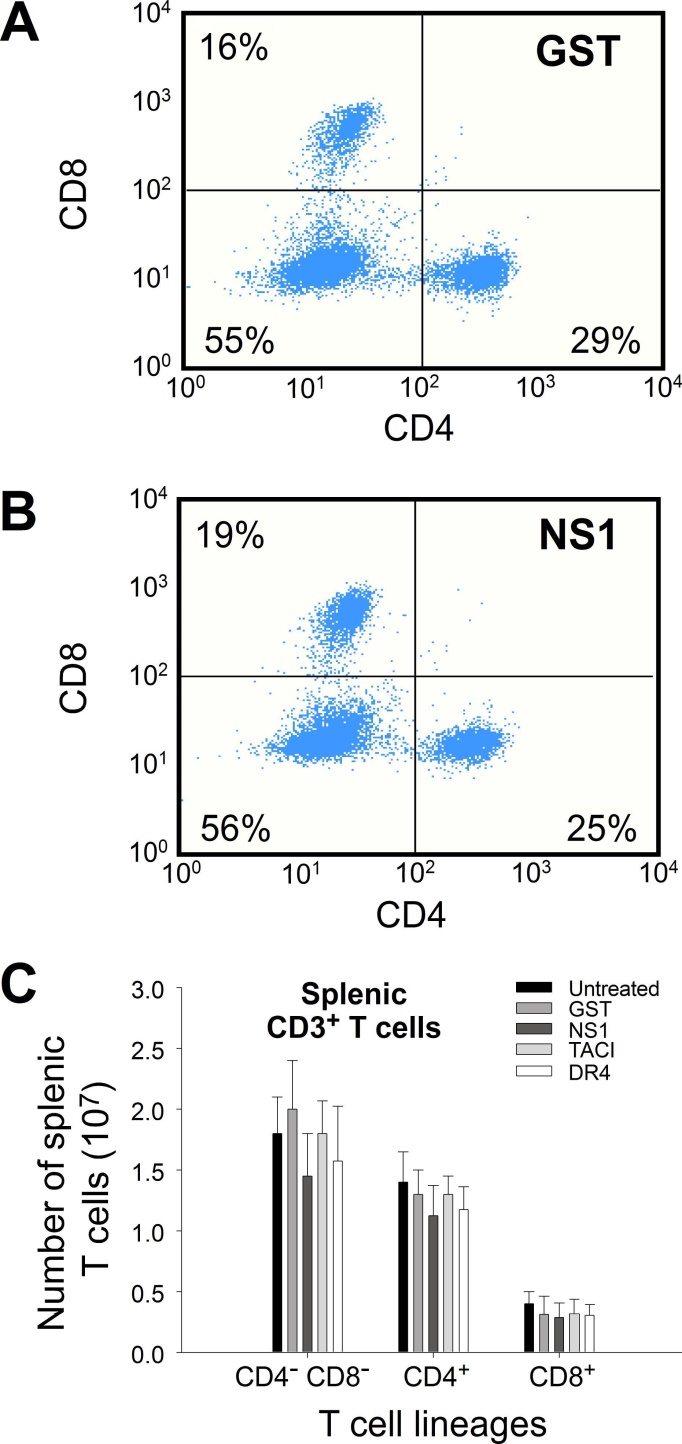


**Suppl. Fig. 5. Mouse T cells subset analyses after immunization with GST, NS1, TACI and DR4 recombinant proteins.** Flow cytometry analysis of T cell populations using CD3, CD4 and CD8 markers was performed after mice were immunized with GST, NS1, TACI and DR4 recombinant proteins. Example graphs were showed (A and B; CD3 gated). Quantification analyses revealed that CD4^+^ and CD8^+^ T cell populations in the spleen were not markedly changed after immunizations with aforementioned recombinant proteins (C). n = 6, 3 independent experiments with 2 replicates.

**Suppl. Fig. 6**


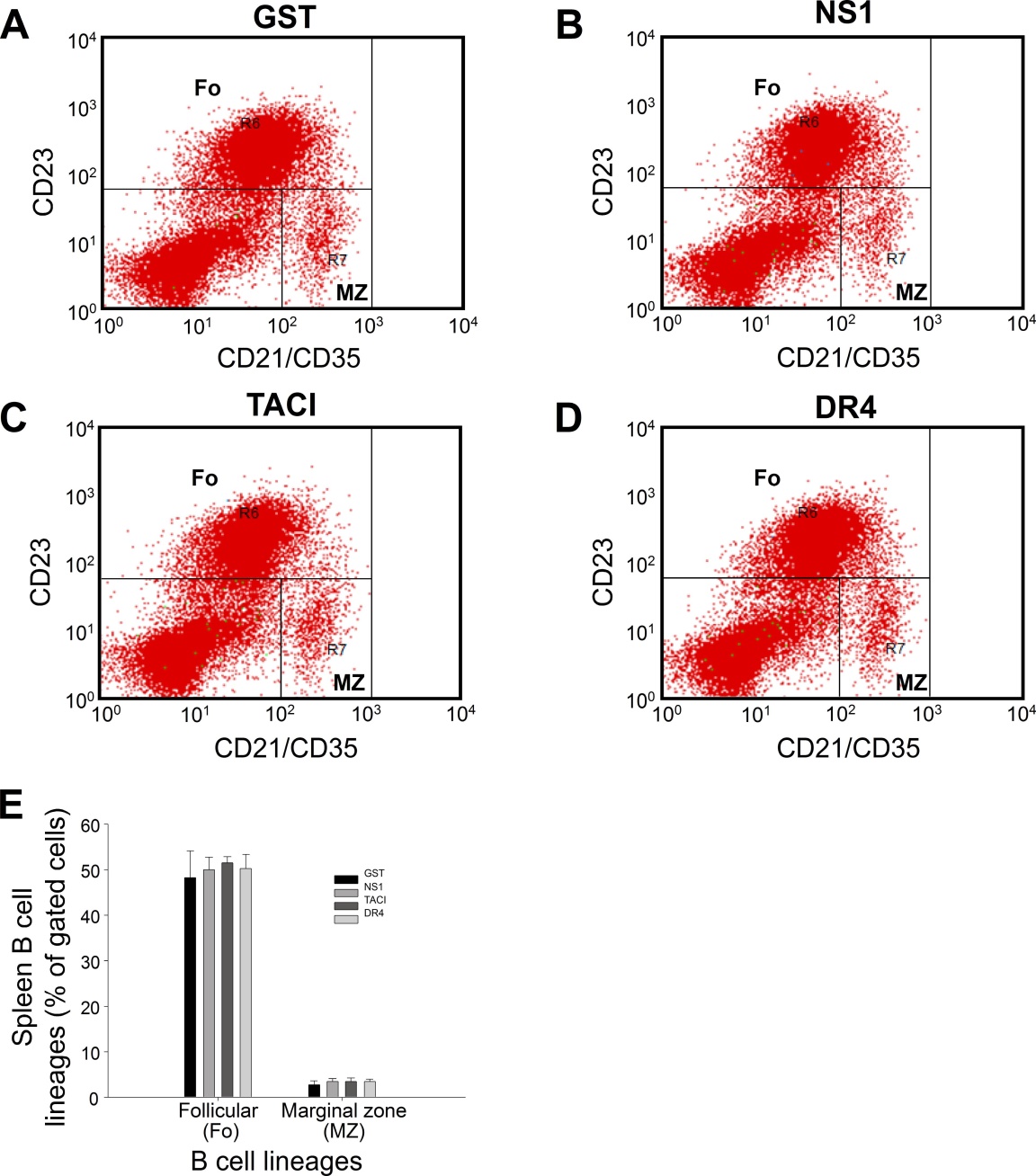


**Suppl. Fig. 6. Mouse splenic follicular and marginal B cell subsets after immunizations with GST, NS1, TACI and DR4 recombinant proteins.** Flow cytometry analysis of splenic B cell precursors using CD21/CD35, CD23 markers was performed after mice were immunized with GST (A), NS1 (B), TACI (C) and DR4 (D) recombinant proteins. Quantification analyses revealed that follicular (Fo; CD21/CD35^int^CD23^+^) and marginal zone (MZ; CD21/CD35^hi^CD23^-^) B cell populations in the spleen were not considerably changed after immunizations with GST, NS1, TACI and DR4 recombinant proteins (E). n = 4, 2 independent experiments with 2 replicates.

**Suppl. Fig. 7**


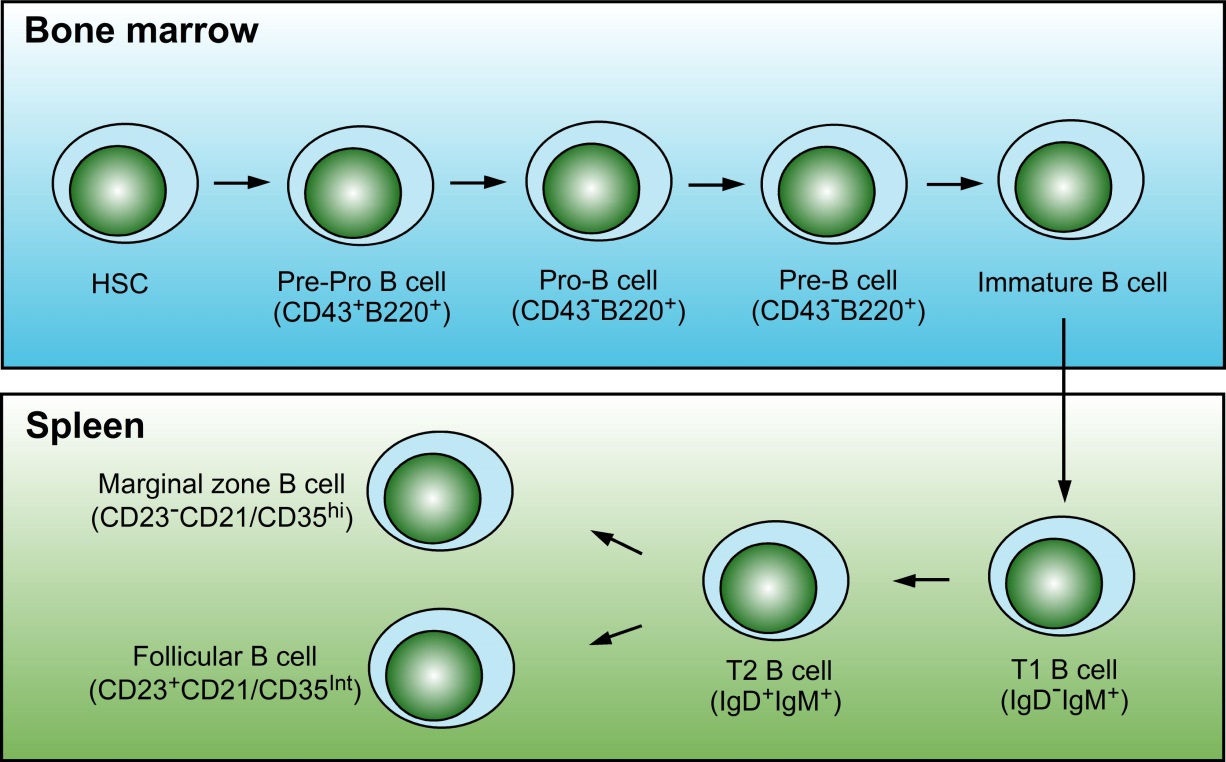


**Suppl. Fig. 7. Development of B cell precursors.** B cell development is illustrated according to previous literatures [1, 2]. B cells firstly develop from hematopoietic stem cells in the bone marrow. After exiting the bone marrow, immature B cells are then further undergoing maturation in the spleen to form naive follicular B cells and marginal zone B cells. HSC, hematopoietic stem cell; pre-B cell, precursor B cell; T1, transitional stage 1; T2, transitional stage 2.

**Suppl. Fig. 8**


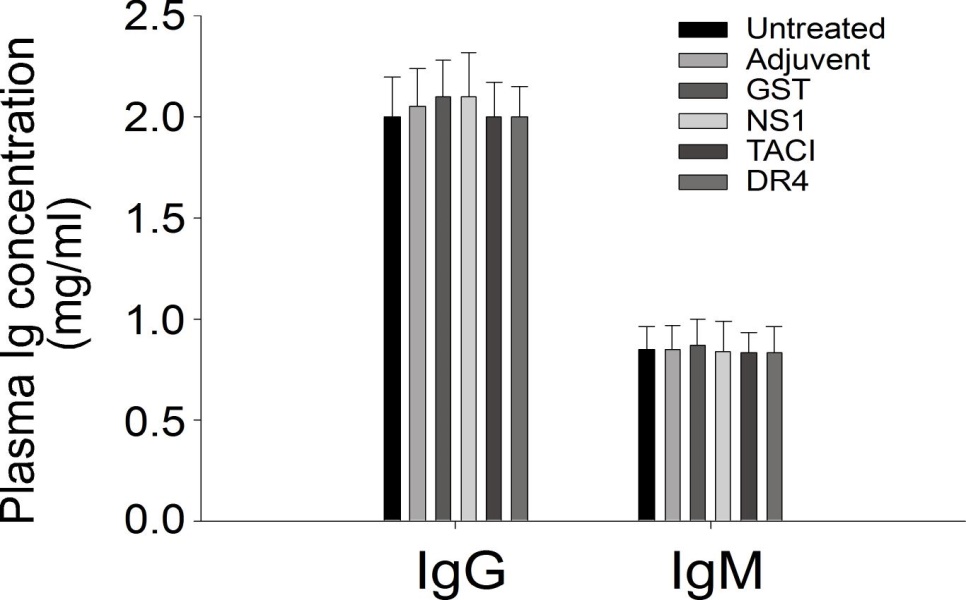


**Suppl. Fig. 8. Total IgM and IgG levels.** The analysis revealed that the total IgG and IgM levels of mice immunized with different proteins did not considerably changed.

**Suppl. Fig. 9**

**
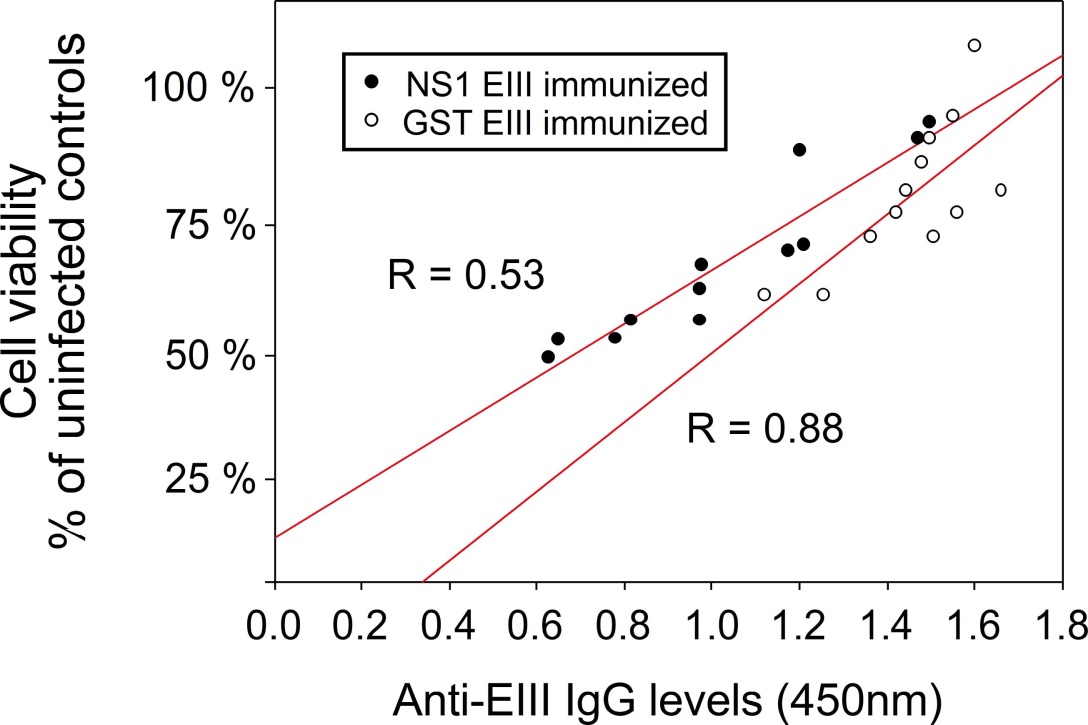
**

**Suppl. Fig. 9. Viability of DENV infected cells are highly correlated to the neutralizing anti-EIII IgG levels.** After NS1 or GST immunizations (2 cycles) and the subsequently EIII immunizations (2 cycles), mouse antiserum was used to perform virus neutralization experiment to rescue DENV-infected BHK-21 cells (20 µL/well of microtiter plate). The ELISA analyzed mouse serum anti-EIII IgG levels, were plotted with cell viabilities of DENV-infected BHK-21 cells. The data revealed that the cellular viability is positively correlated to the serum anti-EIII IgG levels.

**Suppl. Fig. 10**

**
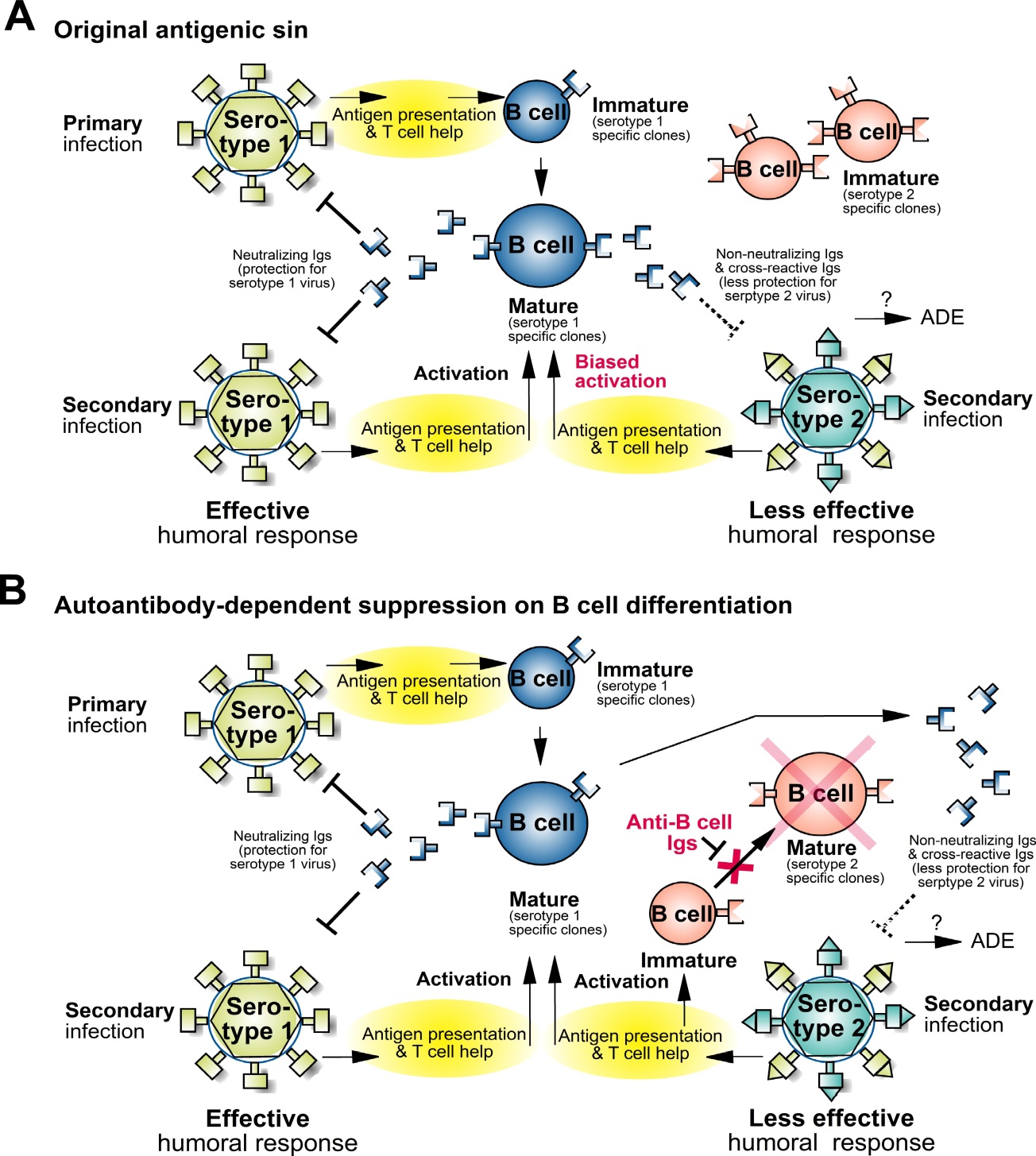
**

**Suppl. Fig. 10. Hypothetical models for humoral immune responses of (A) original antigenic sin, and (B) autoantibody-dependent suppression on B cell differentiation in DENV infections.** In primary infection, antigen of serotype 1 DENV is processed and presented by the antigen presenting cells, leading to the priming and clonal expansion of serotype 1 DENV specific B cells, and neutralizing immunoglobulin (Ig) production.

1. In the condition of original antigenic sin, secondary viral infection leads to a biased stimulation of those serotype 1 virus-specific B and T cells, which will effectively induce protective humoral immune response against secondary serotype 1 virus infection. However, this may result in less effective response against serotype 2 virus infection, because the activation and clonal expansion greatly involve serotype 1-specific but serotype 2 cross-reactive B cells.
2. In the condition of autoantibody-dependent suppression on B cell differentiation, secondary DENV infection leads to an elevation of anti-B cell (dead receptor) Igs, which suppress the maturation of B cell precursors. Effectively protective humoral immune response against secondary serotype 1 virus infection may still effectively induced, as the serotype 1 specific B cells are already matured earlier during the first infection. However, this may result in less effective humoral response against serotype 2 virus infection, because the serotype 2 specific B cells still immature. The maturation of these serotype 2 specific B cell precursors will be blocked by the anti-B cell Igs.

Both hypotheses explain the observation of ineffective humoral response against serotype 2 virus in a secondary infection, and may not be mutually exclusive. The virus and cross-reactive Ig complexes may induce antibody dependent enhancement (ADE)-related pathogenesis to further exacerbate the disease. Arrows: causative connections and activation processes; ⊥ inhibition processes.

**Suppl. Fig. 11**

**
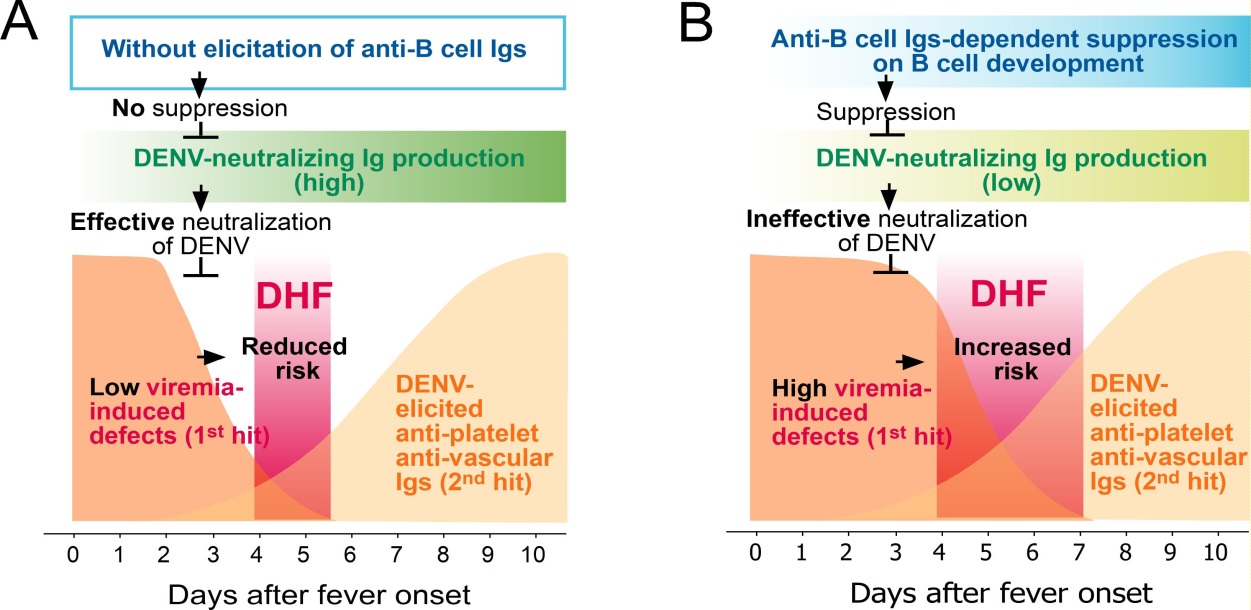
**

**Suppl. Fig. 11. A hypothetical illustration for DENV-induced anti-B cell death receptor Ig fractions on DHF disease progression in the 2-hit model.** A hypothetical model shows the disease progressions influenced without (A), or with (B), B cell death receptor Ig fractions elicitation. Data in this report revealed that elevated levels of these anti-B cell Igs are likely able to suppress the B cell development. This may thereby reduce the quantity and quality of anti-DENV-neutralizing Ig production, and subsequent defense against DENV-induced pathogenesis (A). According to the 2-hit model, DHF hemorrhage pathogenesis can only be induced when both first hit (viremia-induced defects) and second hit (DENV-elicited anti-platelet and anti-endothelial cell Ig fractions) are co-occurring and reached to the threshold [3, 4]. The suppression of B cell maturation and neutralizing Ig production may lead to stronger first hit-induced damages and thus result in an increased risk on the development of DHF (B). Arrows: causative connections. ⊥: Inhibition processes.

**References**

1. Shapiro-Shelef M, Calame K. Regulation of plasma-cell development. Nat Rev Immunol. 2005;5(3):230-42. doi: 10.1038/nri1572. PubMed PMID: 15738953.

2. Nagasawa T. Microenvironmental niches in the bone marrow required for B-cell development. Nat Rev Immunol. 2006;6(2):107-16. doi: 10.1038/nri1780. PubMed PMID: 16491135.

3. Lien TS, Sun DS, Chang CM, Wu CY, Dai MS, Chan H, et al. Dengue virus and antiplatelet autoantibodies synergistically induce haemorrhage through Nlrp3-inflammasome and FcgammaRIII. Thrombosis and haemostasis. 2015;113(5):1060-70. doi: 10.1160/TH14-07-0637. PubMed PMID: 25740324.

4. Sun DS, Chang YC, Lien TS, King CC, Shih YL, Huang HS, et al. Endothelial Cell Sensitization by Death Receptor Fractions of an Anti-Dengue Nonstructural Protein 1 Antibody Induced Plasma Leakage, Coagulopathy, and Mortality in Mice. J Immunol. 2015;195(6):2743-53. doi: 10.4049/jimmunol.1500136. PubMed PMID: 26259584.
